# Clinical-pathological and sociodemographic factors associated with the distant metastasis and overall survival of oral cavity and oropharynx squamous cell carcinoma

**DOI:** 10.4317/medoral.23410

**Published:** 2020-04-09

**Authors:** Paulo Goberlânio de Barros-Silva, Marcela Maria Fontes-Borges, Camila Costa-Dias, José Vitor Mota-Lemos, Maria do Perpétuo Socorro-Saldanha-Cunha, Eric Fernandes-Souza, Thinali Sousa-Dantas, Fabrício Bitu-Sousa

**Affiliations:** 1 Department of Dentistry, Unichristus, Fortaleza, Ceará, Brazil; 2 Ceará Oncology School, Ceará Cancer Institute, Hospital Haroldo Juaçaba, Fortaleza, Ceará, Brazil

## Abstract

**Background:**

The objective of this study was to evaluate the influence of clinical-pathological and sociodemographic factors on the prevalence of distant metastasis (DM) and overall survival in patients with oral cavity and oropharynx squamous cell carcinoma (OOSCC).

**Material and Methods:**

Cross-sectional study based on the records of 404 OOSCC patients evaluated for DM, covering the period 2000-2014. We analysed the influence of age, sex, level of schooling, primary tumor subsite, treatment, marital status, family history of cancer, history of smoking and alcohol consumption, type of health care coverage (private vs. public) and overall survival. Findings were submitted to Fisher’s exact test, Pearson’s chi-squared test, Mantel-Cox log-rank testing and multinomial and Cox regression analysis (SPSS v. 20.0; *p*<0.05).

**Results:**

The prevalence of DM was 5.4% (22/404). The respiratory tract was the most affected DM site (n=9; 40.9%). Male sex (*p*=0.049), oropharyngeal primary tumor (*p*=0.008), stage T3-4 (*p*=0.022), lymph node metastasis (N+) (*p*<0.001) and palliative treatment (*p*=0.005) were directly associated with DM. Patients with oral primary tumours (*p*=0.343) and primary oropharyngeal tumours (*p*=0.242) did not differ significantly with regard to the prevalence of DM. N+ was an independent risk factor for DM (*p*=0.017). Five variables independently reduced overall survival: male sex (*p*=0.035), age >65 years (*p*=0.046), indigenous/brown racial type (*p*=0.045), palliative treatment (*p*=0.035) and DM (*p*=0.048).

**Conclusions:**

Lymph node metastasis independently increased the prevalence of DM and, along with male sex, older age, brown racial type and palliative treatment, was independently associated with poor prognosis in patients with OOSCC.

** Key words:**Neoplasm metastasis, mouth neoplasms, prognosis, carcinoma, squamous cell.

## Introduction

Oral cavity and oropharynx squamous cell carcinoma (OOSCC) have become a major health concern among public health authorities worldwide due to its high incidence, prevalence and mortality (1). Oral cancer is the sixth-most common type of cancer in the world. In Brazil, the Latin American country with the highest prevalence of OOSCC, it is currently the seventh-most common type of cancer (2).

Approximately 90% of neoplasms affecting the oral cavity and oropharynx are squamous cell carcinomas (3). Affected regions include the lips, buccal mucosa, gums, hard palate, tongue, oral floor, tonsils, soft palate, tongue base, salivary glands, retromolar trigone, vallecula, tonsillar arches, palatine tonsils and the posterior and lateral walls of the oropharynx (4).

On average, patients diagnosed with OOSCC survive for 5 years (5). The main factor involved in the onset of OOSCC is smoking (6), but the incidence of OOSCC has also been shown to be associated with human papillomavirus (HPV). In the Brazilian population, men are more susceptible than women, although in general prognosis appears to be better (4). According to the literature, the clinical-pathological profile of patients with OOSCC is typically male sex, age between 50 and 60 years, and history of smoking. The anatomical site most frequently affected by OOSCC is the tongue and oral floor (4).

OOSCC may be treated with radiotherapy, chemotherapy or surgery, or a combination of these. Treatment and prognosis depend on tumor subsite, time of discovery and staging. Prognosis requires stage grouping based on the characteristics of the primary tumor, lymph nodes and systemic metastases (7).

The cervical lymph nodes are the main site of metastasis (7) but, since little has been published on sites of involvement in OOSCC, the possibility of distant metastases (DM) should be considered. The prevalence of OOSCC-related DMs in the lungs and bones ranges from 52% to 83% (8,9). The mean time between symptom onset and detection of the first DM is ~16 months. Once DMs have been identified, the mean survival time is ~5 months (10).

DMs may be detected concurrently with the primary lesion or after treatment (11), and high T and N stages are important risk factors for DM (8). Since the presence of DMs has a strong impact on the prognosis of OOSCC patients (7), the ability to identify risk factors for DM is indispensable for care providers at cancer centers. The objective of the present study was therefore to evaluate the influence of clinical-pathological and sociodemographic factors on the prevalence of DM and overall survival in OOSCC patients.

## Material and Methods

- Type of study and study population

This retrospective study was based on the medical records of 404 OOSCC patients diagnosed and treated at a cancer referral center in Northeastern Brazil (Hospital Haroldo Juaçaba, Ceará Cancer Institute) between 1 January 2000 and 31 December 2014. Only patients with complete TNM clinical staging were included. Patients staged M1 but without identification of the DM site were excluded from the sample. All patient information was retrieved from the institution’s digital cancer registry.

- Study variables

The sociodemographic variables included age, sex, racial type, schooling, marital status, family history, history of smoking and alcohol consumption, and type of health care coverage (public vs. private). The clinical variables included histological type, tumor subsite, TNM staging, tumor staging, and treatment(s) administered. Overall survival (expressed in months) was defined as the time from the beginning of treatment (day/month/year) to death (day/month/year) or the last follow-up visit (day/month/year) (11).

- Statistical analysis

All statistical analyses were performed with the software IBM SPSS Statistics for Windows (v. 20.0), using a 95% confidence interval.

We employed Fisher’s exact test, Pearson’s chi-squared test and multinomial logistic regression modelto evaluate the factors associated with DM and 15-year survival. Kaplan-Meier curves were plotted to determine the mean and standard error of overall survival, which was then submitted to Mantel-Cox log-rank test and Cox regression analysis. All the variables were included in the multivariate models.

## Results

- Clinical and sociodemographic description of metastatic OOSCC

Twenty-two (5.4%) of the 404 OOSCC patients with available TNM staging had metastases. The DMs were located in the trachea/bronchi/lungs (n=9; 40.9%), brain (n=6; 27.3%), bone/joint/articular cartilage (n=5; 22.7%), larynx (n=1; 4.5%) or liver (n=1; 4.5%).

The sample included significantly more males (n=290; 71.8%) than females (n=114; 28.8%). Moreover, males were more prone to develop DMs (*p*=0.049). Segregating the patients by age, 53.0% (n=214) were up to 65 years old and 47.0% (n=190) were over 65, but the two age groups did not differ significantly with regard to the presence of DMs. Most patients (n=256; 63.4%) were of brown racial type, predominantly with incomplete elementary school education (n=99; 31.7%). However, none of the variables above had any influence on the outcome of DM (Table 1).

**Table 1:**
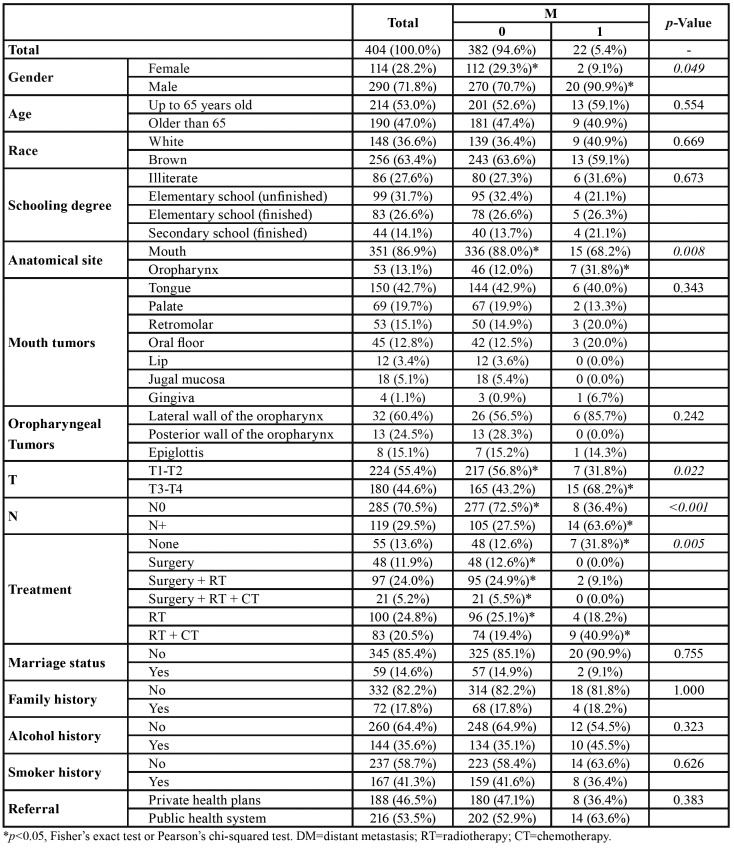
Influence of clinical and sociodemographic characteristics of distant metastases (DM) in patients with squamous cell carcinoma of the mouth and oropharynx (OOSCC) treated at Hospital Haroldo Juaçaba (Ceará Cancer Institute) between 1 January 2000 and 31 December 2014.

The most frequent tumor site was the mouth (n=351; 86.9%), followed by the oropharynx (n=53; 13.1%). Nevertheless, the oropharyngeal region accounted for significantly more cases with DMs (*p*=0.008). Most tumors (55.4%) were classified as T1-T2, but patients with T3-T4 had a higher prevalence of DM (*p*=0.022). No lymph node metastases (N0) were observed in the majority of patients (70.5%), but patients with this finding had a higher prevalence of DM (*p*<0.001) (Table 1).

Radiotherapy was the most frequently administered treatment (n=100; 24.8%), followed by the combination of surgery plus radiotherapy (n=97, 24.0%), and the combination of radiotherapy plus chemotherapy (n=83; 20.5%). Palliative treatment (n=55; 13.6%) and the combination of radiotherapy and chemotherapy (n=83, 20.5%) were significantly associated with DM (*p*=0.005) when compared to other treatment modalities (Table 1).

Most patients were unmarried (n=345; 85.4%) and/or had no family history of cancer (n=332; 82.2%), no history of smoking (n=237; 58.7%) and no history of alcoholism (n=260; 64.4%). Slightly more than half were covered by private health insurance (n=216; 53.5%). None of these variables were significantly associated with the presence of DMs (*p*>0.05) (Table 1).

The most common subsite of oral tumors was the tongue (n=150; 42.7%), while oropharyngeal tumors were most often located in the lateral wall region (n=32; 60.4%). Patients with oral tumors (*p*=0.343) and patients with oropharyngeal tumors (*p*=0.242) did not differ significantly with regard to the prevalence of DMs (Table 1).

Following the multivariate analysis, the only variable which remained independently associated with the presence DMs was lymph node metastasis (*p*=0.017), corresponding to an increase in the prevalence of DM by a factor of 3.16 (Table 2).

**Table 2:**
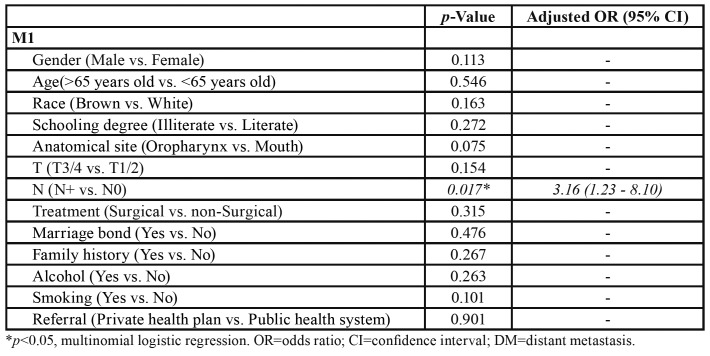
Multivariate analysis of risk factors for distant metastasis (DM) in patients with squamous cell carcinoma of the mouth and oropharynx (OOSCC) treated at Hospital Haroldo Juaçaba (Ceará Cancer Institute) between 1 January 2000 and 31 December 2014.

- Prognostic factors: analysis of 15-year survival

**Figure 1:**
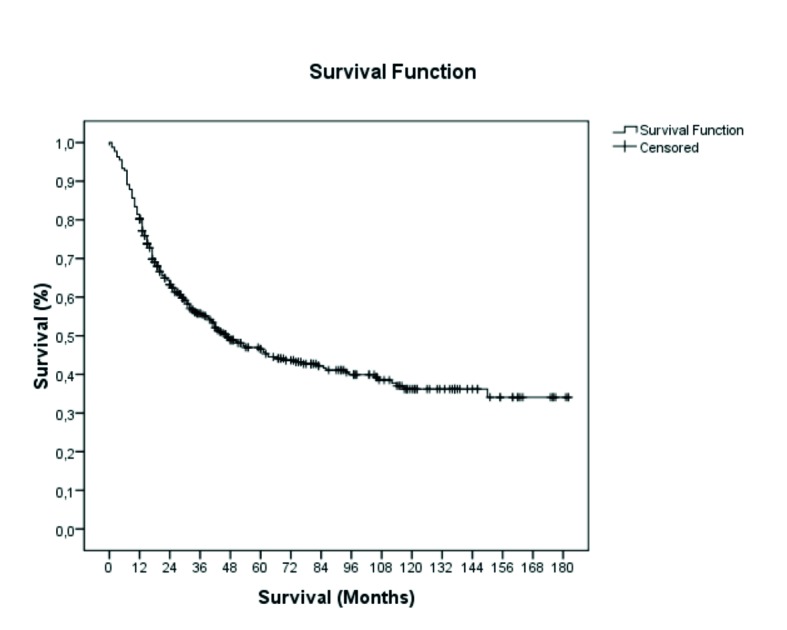
15-year follow-up and global survival of patients with squamous cell carcinoma of the oral cavity and oropharynx (OOSCC) treated at Hospital Haroldo Juaçaba (Ceará Cancer Institute) between 1 January 2000 and 31 December 2014 (Kaplan-Meier method).

After 15 years of evaluation, the survival rate was 46.8% (n=189) and the mean overall survival was 85 ± 4 months. Half the patients (median) had died 47 months into the study period (Fig. 1).

The overall survival prevalence and the mean survival time were significantly better in female patients (*p*=0.001; *p*=0.006), in younger patients (≤65 years) (*p*=0.018; *p*=0.024) and in patients of white racial type (*p*=0.043; *p*=0.039), respectively. The level of schooling influenced the overall 15-year survival: the rate was higher for patients with complete elementary or high school education (*p*=0.049), but the mean survival time did not vary significantly (*p*=0.626). Likewise, survival rates were higher for patients with oropharyngeal tumors than for patients with oral tumors (*p*=0.007), with no difference in mean survival time (*p*=0.087) (Table 3).

**Table 3:**
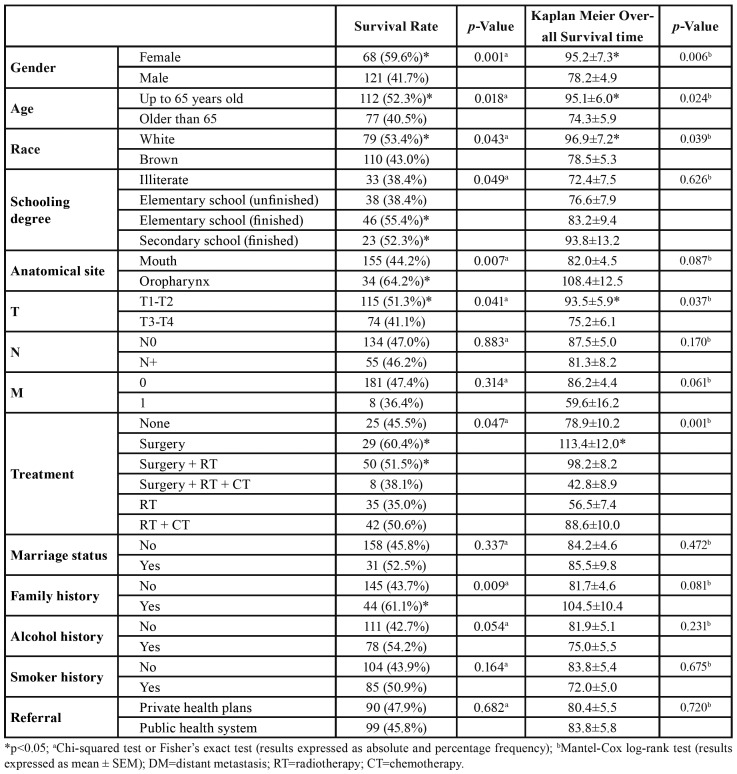
Influence of clinical and sociodemographic variables on the overall survival of patients with squamous cell carcinoma of the mouth and oropharynx (OOSCC) treated at Hospital Haroldo Juaçaba (Ceará Cancer Institute) between 1 January 2000 and 31 December 2014.

Patients with T1-T2 tumors had a higher survival rate (*p*=0.041) and a longer survival time (*p*=0.037) than did patients with T3-T4 tumors. The presence of lymph node metastasis and DM was not significantly associated with survival rate or mean survival time (*p*>0.05). As for treatment form, survival rate (*p*=0.047) and survival time (*p*=0.001) were better for patients treated with surgery or with a combination of surgery and radiotherapy than for patients who received other treatment modalities (Table 3).

Marital status, history of alcohol consumption and smoking, and type of health care coverage had no significant influence on survival rate or time (Table 3). The survival rate was higher for patients with than without family history of cancer (*p*=0.009).

In the multivariate analysis, five variables independently reduced overall survival: male sex (HR=1.55; *p*=0.035), age >65 years (HR=1.39; *p*=0.046), brown racial type (HR=1.42; *p*=0.045), non-surgical treatment (HR=1.11; *p*=0.035) and DM (HR=1.71; *p*=0.048), which displayed the highest hazard risk (Table 4).

**Table 4:**
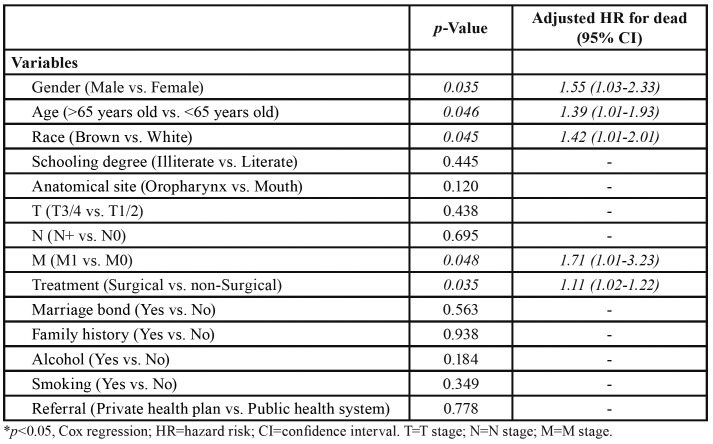
Multivariate analysis and independent risk factors for poor survival in patients with squamous cell carcinoma of the oral cavity and oropharynx (OOSCC) treated at Hospital Haroldo Juaçaba (Ceará Cancer Institute) between 1 January 2000 and 31 December 2014.

## Discussion

In the present study, we drew the clinical-pathological and sociodemographic profile of 404 OOSCC patients evaluated for the presence of DM. The prevalence of DM was 5.5%, matching findings in the literature, although percentages may be underestimated depending on sample size. Thus, in a study involving over 1,000 patients, the prevalence of DM was 9.6%. Variations in prevalence may also depend on whether the mouth and oropharynx were evaluated together, as in this study (9).

DM affected the lower airways in 40.9% of our patients. Another observational study found 50% of DMs to involve the lungs, making that structure the main target. Although sites other than the lungs were affected in more than half our patients with DM, the lungs appear to be the primary site of DM involvement (10). The next-most prevalent sites of OOSCC-related DM were the central nervous system (27.3%) and bone tissue (22.7%). In another retrospective study, DMs targeted bone tissue in 34% of cases, indicating the need for bone evaluation in patients with high staging (8), especially since stage T3 and T4 tumors are associated with higher prevalence of DM.

Previous studies have shown tumor extension to be independently associated with the risk of DM (12), especially for tumors in the hypopharynx and supraglottis (9). In our sample, patients with oropharyngeal tumors were more likely to have DM than patients with oral tumors, supporting the notion that the presence of primary tumors in the lower neck structures is a risk factor for DM (10). The greater likelihood of DMs in patients with oropharyngeal tumors may be explained by the greater difficulty of evaluating the lower neck area for primary tumors, but it should also be kept in mind that the lower structures are more vascularized and have better lymph node connectivity (10). In our study, the presence of lymph node metastases was an independent risk factor for DM, suggesting that lymph node involvement may contribute to disease dissemination. Lymph node involvement is reported to be less frequent in HPV-related OOSCC (4). We did not evaluate HPV infection in this study, but the strong association observed between the oropharynx and DM points to other risk factors, such as smoking and alcohol consumption.

The fact that approximately 50% of patients with head and neck SCC have positive lymph nodes at diagnosis shows the need for DM investigation, especially since the presence of DMs significantly reduces survival (13-15). Lymph node involvement is the most important risk factor for OOSCC recurrence (7) and in this study was the only risk factor for DM.

Another risk factor for DM is male sex. This is consistent with previous studies (8), although the distribution seems to depend on the population evaluated (10). Perea *et al*. suggest the distribution is influenced by the timeliness of diagnosis, which in many settings occurs earlier in women (16).

As shown above, the most prevalent subsites were the tongue (oral tumors) and the lateral wall (oropharyngeal tumors), but the subsite had no significant influence on the prevalence of DM. This diverges from the results of another study showing that the lower and more posterior the subsite, the higher the risk of DM (9).

In this study, the 15-year survival rate of patients with OOSCC was 46.8%. This is somewhat better than would be expected based on the results of Moro *et al*. who registered survival rates of 42% and 38% at 5 and 10 years, respectively (5), and Köhler *et al*. who reported 53.42% overall survival for a sample with a mean follow-up of 44 months (7). Prognosis is even worse in some populations: in southern Thailand the survival rate was 24.1% at 5 years and 25.95% at 10 years. The authors attributed these Figures to the more advanced stage at which their patients were diagnosed and to the treatments administered (17).

Socioeconomic status plays a key role in survival statistics. Most of our patients were covered by private health insurance (potentially favoring survival), but in earlier investigations on similar populations conducted by our group, the level of schooling and history of smoking were the main determinants of poor prognosis (11,18). Individuals of low socioeconomic status tend to have limited access to health services and to be less aware of risk factors, which in turn increases the risk of late prognosis and shortens survival. More effective public health policies are required to change this unfortunate scenario characteristic of countries with high income concentration.

The main determinants of good prognosis in our sample were female sex, age <65 years and complete high school education. This is compatible with previous studies arguing that men are generally more prone to smoking and alcoholism. Low levels of schooling may be associated with lack of health awareness, limited access to health services, increased risk of malnutrition, and greater exposure to OOSCC risk factors (4,10,19).

Some authors have concluded that marital status has a significant impact on the prognosis of OOSCC (20), but no such association was observed in our study. In contrast, in our sample the protective factors were female sex, age <65 years, white racial type, high level of schooling, oropharyngeal subsite, T1-T2 staging, and surgical treatment. However, in the multivariate analysis, only male sex, older age, brown racial type, palliative treatment, and DM were associated with poor prognosis, with DM presenting the highest risk of death.

Thus, despite the limited sample size and retrospective nature of our study, we observed that male patients with oropharyngeal cancer classified as N+ present a higher prevalence of DM, which is the factor most strongly associated with poor overall survival. Based on this, we recommend submitting patients with N+ OOSCC to systemic evaluation for early detection of DMs, thereby potentially improving prognosis.
